# 

**Published:** 1867-05

**Authors:** 


### CONTENTS.

ORIGINAL COMMUNICATIONS.

Function, .......... 189
A growl from an Ignorant Man Against some Scientific Technicalities, .......... 198

PROCEEDINGS OF SOCIETIES.

Extracts from Discussions of the Mississippi Valley Association of Dental Surgeons, .......... 203
Communications, .......... 230, 231

EDITORIAL.

Dental Teaching, .......... 232
The Canines, .......... 233
Cohesive Shred Gold, .......... 233
Dental Instruments, .......... 234

